# Barriers and facilitators to implementing evidence based bleeding management in Australian Cardiac Surgery Units: a qualitative interview study analysed with the theoretical domains framework and COM-B model

**DOI:** 10.1186/s12913-021-06269-8

**Published:** 2021-06-05

**Authors:** Bronwyn L. Pearse, Samantha Keogh, Claire M. Rickard, Yoke L. Fung

**Affiliations:** 1 School of Nursing and Midwifery, Griffith University, Brisbane, QLD Australia; 2 Departments of Surgery, Anaesthesia and Critical Care, The Prince Charles Hospital, Sippy Downs, QLD Australia; 3 School of Health & Sports Sciences, University of Sunshine Coast, Sippy Downs, Australia; 4 School of Nursing and Centre for Healthcare Transformation, Queensland University of Technology, Kelvin Grove, QLD Australia; 5 Alliance for Vascular Access Teaching and Research, Menzies Health Institute Queensland, Griffith University, Brisbane, Australia

**Keywords:** Cardiac surgery, Australia, Bleeding, Barriers, Facilitators, Theoretical domains framework, COM-B model

## Abstract

**Background:**

Bleeding during cardiac surgery is a common complication that often requires the transfusion of blood products. The combination of bleeding and blood product transfusion incrementally increases adverse outcomes including infection and mortality. Following bleeding management guideline recommendations could assist with minimising risk but adherence is not high, and the cause for lack of adherence is not well understood. This study aimed to identify barriers and facilitators to practicing and implementing evidenced-based intra-operative, bleeding management in Australian cardiac surgery units.

**Methods:**

We used a qualitative descriptive design to conduct semi-structured interviews with Australian cardiac surgeons, anaesthetists and perfusionists. The Theoretical Domains Framework (TDF) was utilised to guide interviews and thematically analyse the data. Categorised data were then linked with the three key domains of the COM-B model (capability, opportunity, motivation - behaviour) to explore and understand behaviour.

**Results:**

Seventeen interviews were completed. Nine of the 14 TDF domains emerged as significant. Analysis revealed key themes to improving capability included, standardisation, monitoring, auditing and feedback of data and cross discipline training. Opportunity for change was improved with interpersonal and interdepartmental collaboration through shared goals, and more efficient and supportive processes allowing clinicians to navigate unfamiliar business and financial models of health care. Results suggest as individuals, clinicians had the motivation to make change and healthcare organisations have an obligation and a responsibility to partner with clinicians to support change and improve goal directed best practice.

**Conclusion:**

Using a theory-based approach it was possible to identify factors which may be positively or negatively influence clinicians ability to implement best practice bleeding management in Australian cardiac surgical units.

**Supplementary Information:**

The online version contains supplementary material available at 10.1186/s12913-021-06269-8.

## Background

Excessive bleeding occurs during and after cardiac surgery in up to 10% of patients; and the most common treatment is the transfusion of blood products [1, 2]. However, blood transfusion is a form of tissue allotransplantation which may be associated with circulatory overload, acute lung injury, febrile and allergic and haemolytic reactions, alloimmunisation, immunomodulatory effects, and bacterial and infectious disease transmission [3–6]. Furthermore, the combination of excessive bleeding, management with blood transfusion and re-exploration surgery incrementally increases adverse outcomes including infection and mortality [6–13].

Many evidence-based, guideline-supported strategies exist to manage bleeding, including the collaborative contribution of the surgical team [14–18]. In 2010, the implementation of multidisciplinary and multimodal concepts to manage bleeding and blood loss were endorsed by the World Health Organisation (WHO) as part of the Patient Blood Management paradigm (PBM) [19]. However, the complex and dynamic context of cardiac surgery makes the implementation of bleeding management a genuine challenge for the surgical team. Complex patients requiring individualised and co-ordinated care from numerous specialties in the same physical space, at the same time, requires active collaboration. The significant variation in bleeding management practice and outcomes noted in literature may be a result of these managing these challenges [1, 20–23].

Although clinicians are expected to implement strategies that provide best outcomes, the implementation, quality assurance and quality improvement knowledge and skills that are required to change practice are often not a core component of clinical training [24]. Difficulties with implementation may be compounded by factors relating to the environment, context, resources, and social influences [25–27]. As a result, clinicians may not always provide evidence-based bleeding management, even when they perceive patients would benefit from such interventions.

It is known from behavioural theories that clinicians must have the capability to improve practice or make change, as well as the opportunity and motivation to do so [28]. In the cardiac surgical setting, the barriers and facilitators to the implementation and provision of evidence-based bleeding management are unclear. Previous research has focused on outcomes of bleeding including the transfusion of blood products rather than focussing upstream, investigating management of the bleeding episode. Consequently, there are important gaps in knowledge regarding clinicians’ ability to implement and provide best practice bleeding management. It is therefore necessary to investigate and understand more about this area of practice and the implications on health care outcomes. The aim of this qualitative study was to identify barriers and facilitators that surgeons, anaesthetists and perfusionists face when practicing and implementing evidenced-based, intra-operative bleeding management in Australian cardiac surgery units.

## Methods

### Design

This study employed a qualitative design to investigate the perceptions of barriers and facilitators to practicing and implementing bleeding management in Australian cardiac surgery units. Semi-structured interviews with cardiac surgeons, cardiac anaesthetists and perfusionists were undertaken in November and December 2018.

### Theoretical framework

We employed two theoretical models of behaviour change to provide both theoretical and pragmatic guidance for this study: 1. the Theoretical Domains Framework (TDF), and 2. the Capability, Opportunity, and Motivation Behaviour Model (COM-B) [29]. The TDF is a framework that can be used in a theory based evaluation to identify factors (i.e., barriers and facilitators) that may influence behaviour. The TDF is a ‘first step’ to identify and categorise a given ‘barrier and/or facilitator’ to behaviour rather than an explanation of how change takes place. The 14 validated TDF domains are: 1. knowledge, 2. skills, 3. social/professional role and identity, 4. beliefs about capabilities, 5. optimism, 6. beliefs about consequences, 7. reinforcement, 8. intentions, 9. goals, 10. memory, attention, and decision processes, 11. environmental context and resources, 12. social influences, 13.emotion, and 14. behavioural regulation. The interview topic guide was based on the 14 domains to view and categorise cognitive, affective, and social and environmental influences on behaviour. The TDF-based interview topic guide was developed for this study, refined through discussion with the study team and pilot tested with a surgeon, an anaesthesiologist and perfusionist. (Additional file 1).

**Fig. 1:**
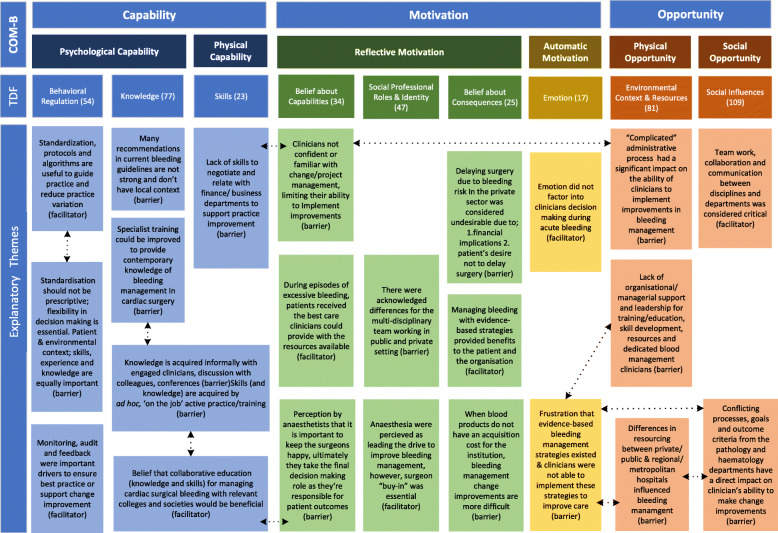
Explanatory themes describing the barriers and facilitators to practicing and implementing evidence-based bleeding management in Australian Cardiac Surgery Units

Open-ended questions were used, and the number of questions ranged from one to three for each TDF domain. The same questions were used with for all interviewees with follow-up prompts and probing questions included when necessary to address specific constructs within the domains. Data categorised with the TDF framework were distilled and linked with the three key domains of the COM-B model, then used to analyse and understand behaviour. The COM-B model of behaviour can be used to identify what needs to shift or be modified, so that behaviour change interventions can be effective. The model identifies three components that need to be present for behaviour to occur: capability, opportunity, and motivation. Capability covers a person’s psychological and physical capacity to adopt a particular behaviour. Opportunity relates to physical and social opportunities that influence behaviour. Motivation covers the thought processes that direct behaviour. Both capability and opportunity can influence motivation. Therefore, while motivation addresses whether clinicians *will or won’t* adopt a particular behaviour; capability, and opportunity address whether a clinician ‘*can or can’t* adopt it. These components are dynamic and interact over time where behaviour can be seen as part of a system with positive and negative feedback loops [30–32].

### Participants

Participants who took part in the study were those clinicians involved in the direct management of intra-operative bleeding and included cardiac surgeons, cardiac anaesthesiologists and clinical perfusionists. A combination of convenience and snowball sampling techniques were used to recruit 17 clinicians from a previous national cross-sectional survey on bleeding management practice by the same authors [33]. Participants from that survey were asked to email their interest in participating in a future qualitative interview study and participants emerged through a process of reference. Based on the threshold for data saturation in previous studies with health care clinicians and using the TDF, we projected that 12 to 18 interviews would be required [34]. Final sample size was again determined through data saturation, which was considered to have occurred when no new data were identified in three successive interviews. Invitation letters, study information sheets and a consent form were sent via email inviting 23 clinicians to participate in interviews. If no response was received after a week, a second email was sent. The primary reason for non-participation was lack of time or scheduling issues.

### Data collection

Brief introductions took place before the consent that was emailed to participants at recruitment was reviewed, and signed prior to the interview. In depth interviews were conducted by the lead author and were audio recorded. Open-ended questions were used to encourage participants to explore their experiences and specific instances with managing, or implementing bleeding management, including barriers and facilitators [35].

### Data analysis

For the purpose of consistent coding, a coding guide was developed based on the published definitions and concepts of the TDF domains. (Additional file 2) The 17 audio-recorded interviews were transcribed verbatim and imported into the qualitative data analysis software package NVivo 12 (QSR International). Excerpts were coded into the main domains of the TDF using theory-based content analysis by the first author (BP). In the next stage, specific beliefs, including both barriers and facilitators were collated, defined, and grouped into sub-themes under each domain. The NVivo software organises data into the number of references or occurrences attributable to participants. Further analyses were also performed to compare interviewees’ statements by hospital type (public/private, metropolitan/regional). A second and third member (YLF and SK) independently analysed the interviews (approximately 65 and 35% respectively), to ensure reliability of the coding guide. All coding was discussed and agreed upon by the study team. The research team met several times to refine categories and clarify any issues. Memos were used to record relevant discussions and coding notes.

Criteria were developed to determine which domains of the TDF were ‘relevant’: 1. qualitatively; where specific beliefs were coded frequently within a domain and 2. quantitatively; where domains contained strong beliefs regarding barriers/facilitators to practicing and implementing provide evidence-based bleeding management. Relevance was considered to be achieved when domains met both criteria. Following the data analyses, all participants were given the opportunity to review the synthesised member check document. Member checking provided participants with the opportunity to add clarification or new information and prioritise the inferences, emerging concepts, and initial themes.

## Results

Participants (cardiac surgeons, *n* = 5, cardiac anaesthesiologists, *n* = 7, clinical perfusionists, n = 5) represented key stakeholders involved in practicing and implementing bleeding management in cardiac surgery in Australia. Participants reported practicing from 9 to 29 years, and practiced across different states and settings including public (6), private (1) and both (10). Length of interviews ranged from 27 min to 53 min. Specific participant demographics or any identifying data within the quotes were not included to protect anonymity.

Of the 14 TDF domains, nine emerged as significant to categorise clinicians’ beliefs about the barriers and facilitators to managing bleeding and implementing change to improve practice. Four TDF domains were present but rare. Reinforcement was not idenified as a domain (Table 1).

**Table 1:** TDF Domains reported by occurrence and participants

COM-B	TDF	No. of Occurrences	No. of Participants	% Participants
***Relevant Domains***				
Capability	Behavioural Regulation	54	14	82%
	Knowledge	77	13	76%
	Skills	23	12	71%
Opportunity	Environmental Context and Resources	81	17	100%
	Social Influences	109	16	94%
Motivation	Belief about Capabilities	34	14	82%
	Social Professional Roles & Responsibility	47	14	82%
	Belief about Consequences	25	12	71%
	Emotion	17	10	59%
***Rarely Reported Domains***				
Capability	Memory, Attention, and Decision Making	4	4	23%
Motivation	Intentions	4	4	23%
	Goals	5	3	18%
	Optimism	7	3	18%

A number of explanatory themes (barriers and facilitators) were connected within and across domains, while remaining a specific theme (illustrated by connecting arrows in Fig. 1)

### Capability: psychological capability, physical capability

‘Capability’ (COM-B) can be explained as the clinician’s capacity to engage in the management of actual bleeding episodes or implement practice improvement. Six barriers and three facilitators emerged in relation to this construct.

#### Behavioural regulation (psychological capability)

These included three related to “Behavioural Regulation” (TDF) whereby clinicians described standardisation with protocols and decision support tools as useful to guide and reduce variation in practice. All quotations are followed by recognition of profession (S) for surgeon, (A) anaesthetist, (P) perfusionist.

*“I think that protocol-based practice has a huge amount going for it – reproducibility of what you do, patient safety, everyone being able to be on the same page every time. I think that the answer to a lot of these problems, is having protocols to drive your practice” (A)*

*“So, to follow a protocol and get the majority of problems (bleeding) sorted – 95-99% of the time, is really easy” (A)*

*“If it’s done every time, it’s part of the vernacular … . it becomes part of the language. It has to become part of the standard practice, so it’s more habit actually, that changes” (A)*

*“An algorithmic guideline helps you remember things that you may have otherwise forgotten” (A)*

However, there was also the belief that flexibility in decision making using experience were equally important because of locally contextual clinical and environmental issues.

*“It’s a combination of evidence based and personal preference and personal experience” (S)*

*“It’s never going to be a rigid application of guideline for every single patient and there has to be that of course” (A)*

*“It’s like a mental check list but it’s dependent on the patient, the procedure, where I’m operating and who I’m working with” (S)*

*“One of the problems with clinical medicine is that people do tend to think dichotomously rather than continuously and that’s not helpful” (A)*

Additionally, participations considered behaviours and the ability to implement improvement could be influenced by audit and feedback. This was considered particularly relevant as surgeons and anaesthetists were highly driven, often competitive, and capitalising on these traits was considered an enabler for practice improvement.

*“It’s about incentive, we have brought it in for them … … . the de-identified data, where they all want to be like everybody else. They don’t want to be an outlier, so when you can present them with a graph with their de-identified, they take note” (A)*

#### Knowledge (psychological capability) and skills (physical capability)

Four themes emerged related to the TDF domain ‘*knowledge’,* two related to *‘skills’* specifically and two themes bridging both domains. (Table 1) In the first bridged theme, participants supported the concept of joint educational opportunities with relevant colleges and societies to improve both *‘knowledge’* and *‘skills’* within the multidisciplinary framework*.* There was a belief that this type of joint training could address the lack of a common language.

*“Collaborative teaching would be helpful. It has to be multi-disciplinary though, so you can’t have the cardiac ANZSCTS* (Australian & New Zealand Society of Cardiothoracic Surgeons) *doing one thing for the cardiac surgeons and ANZCA’s* (Australia and New Zealand College of Anaesthetists) *cardiac special interest group doing something for the cardiac anaesthetists and they’re different. You’ve got to be working from the same knowledge base” (A)*

*“An area where both groups can get together and learn and have a combined approach. I think team management is important so that when we say to the surgeon, X and Y are ok but maybe you should consider Z, but they say to us but A, B and C. We’re both talking the same language and making a collective decision based on that” (A)*

In the second bridged theme participants overwhelmingly reported *‘skills’* and *‘knowledge’* were acquired informally by clinicians with a particular interest in bleeding management, then learnings passed in an ad hoc way or, on the job discussion.

*“Peripherally, there are bits of knowledge you can gain and obtain, but I’m not aware of a specific course or a specific online teaching resource for bleeding in cardiac surgery” (A)*

*“Currently you have knowledge that is dispensed by individuals, it is uncoordinated, based on opinion, and I think that is part of the confusion with blood management” (P)*

*“I do think that the bleeding that you see in cardiac surgery is unique and the patterns of coagulopathy that you see associated with cardiac surgery are unique and that trying to lump those in with other major bleeding and other surgery or trauma is a mistake” (A)*

The majority (but not all) participants believed that specialist training could be improved with the inclusion of more up to date learning for example, related to the cell-based model of coagulation, viscoelastic haemostatic assays and goal directed therapy.

*“I never had any formal teaching in haemostasis except those diagrams in medical school that actually mean nothing, the INR and PT and APTT. I don’t think any of those tests are useful in bleeding management” (S)*

*“I think it needs to get into the training programs. It needs to become second nature for people. We’re sort of attacking it from the wrong end trying to grab people by the time they're out and invested in their current practice” (A)*

*“I think there is probably a very variable range of knowledge among cardiac anaesthetists about management of coagulation, to be honest with you” (A)*

*“In my training, I got taught very little about bleeding. Not at medical school and not at surgical school, so when we started doing blood management here, I knew a bit about bleeding and haemostasis management but I didn’t know a lot and I didn’t know anything about ROTEM or TEG or any of those technologies and so I had to learn” (S)*

*“Every cardiac surgeon during its training is well-experienced. I think in six years they know how to control bleeding and how to manage patients with bleeding” (S)*

*“Anaesthetists probably have the most education on managing major haemorrhage and bleeding, we have to do major haemorrhage and critical bleeding modules for CPD (continuing professional development)” (A)*

*“ … they are still being taught these old pathways that exist in test tubes and not being taught practical stuff in terms of bleeding management” (S)*

A barrier to ‘*knowledge’* was widespread acknowledgement of non-compliance with current guidelines. Deviations from recommendations were considered an accepted part of practice. It was generally believed this was due to divergent recommendations, recommendations based on low levels of evidence, and a lack of local relevance.

*“I am aware of multiple sets of guidelines through my own work and research. They don’t all harmonise of course, and so that’s problematic” (A)*

*“They* (Australian & New Zealand Society of Cardiothoracic Surgeons) *would be an authority that I would turn to because they’re local, but they don’t have anything specific” (A)*

*“The challenge that we have for example, the European guidelines, there is a lot of factor concentrates and they have different systems, so they don’t necessarily apply to here” (A)*

*“The problem being of course is that they (guidelines) are disparate in some respects. On occasions, the guidelines actually contradict each other, or else they have different weightings for the level of evidence that they present and that of course is a problem” (A)*

Participants felt that they often lacked the skills needed to negotiate and relate with finance/business departments when trying to introduce new equipment, testing, products, or additional clinical time to support practice improvement. It was felt that, not only the language used by clinicians, but the goals and outcomes of these different departments did not align and was a source of frustration.

*“In the public hospital system, a huge barrier is that doctors and medical people are not trained in the same way in business and in budgeting and even how to interact with groups outside the medical profession” (A)*

*“The project management skills that you need to set up a program are quite significant, and I think many of us, not only don’t have the time to do it, but don’t have the skills unless you have them intrinsically, they are not there” (A)*

*“You’ve got to learn how to talk to all these other people, the funders and the bean counters, you’ve got to learn to talk to them in a way that they understand and sell yourself” (A)*

### Opportunity: physical opportunity, social opportunity

‘Opportunity’ (COM-B) can be explained as all the factors that lie beyond the individual, that make the management of bleeding or implementation of practice improvement, possible. These included factors in the environment that encourage or discourage these goals. These factors can be physical for example, time constraints, resources, cost, physical environmental barriers. Or they can be related to social context of practice including interpersonal, intradepartmental, or interdepartmental influences, group or individual attitudes, culture, or the expectations of others. Five barriers and one enabler emerged as influencing participants within this construct. These included three related to three related to ‘Environmental context and resources’ and two related to ‘Social influences’.

### Environmental context and resources (physical opportunity)

Participants believed that complicated administrative processes negatively influenced their ability to implement improvements in bleeding management. Compounding these barriers were a lack of organisational and/or managerial support and leadership for training/education, skill development, resources, and dedicated blood management clinicians. Participants also reported considerable time restraints due to their primary clinical responsibility with little or no time to dedicate to write business cases for funding, or develop processes, policies, tools, and educational packages required to change practice.

*“We’ve got pain nurses and infection-control nurses, we’ve got joint-care nurses, we’ve got colostomy nurses, but we don’t have a blood management nurse who is here every day like those others, who can do this stuff. I’ll know that there is an issue but it’s going to take me two weeks until I can actually sit down and make an attempt to address it. A person who had the time, dedicated to this sort of thing, would really make a big difference” (A)*

*“Putting a business case together, no body teaches you how to do that and it takes a lot of time. We're clinicians looking after patients, who has the time?” (A)*

*“You need to have both support from the Chief Executive as well as the individual people in the room. I think all are very important” (S)*

*“Administrative bureaucracy that prevents improvements from happening. That’s one thing you have to overcome. I think that’s definitely a big barrier, it’s frustrating” (A)*

*“The external factors, non-clinical entities, which affect us, I don’t think that anybody would stand in the way of us delivering evidence-based care on a day to day basis, but they will stand in the way of providing the resources that we need to do it over the short to medium long term” (A)*

Participants agreed that differences in resourcing between private/public & regional/ metropolitan hospitals influenced bleeding management and the ability to implement practice improve initiatives.

*“It’s difficult, I can’t really say, ‘let’s not give anything’ because we don’t have any evidence without a ROTEM or access to platelet function, and I am not the one who has a bleeding patient under my hands. I think we have some power, to discuss and talk but no real time information to make treatment decisions and that limits our input” (A)*

*“I don’t have a good answer for how it would work in the private sector, but the public sector you have a chance of getting a paid person, generally like a nurse educator type role, that works best I think in the public sector” (A)*

*“ … .. it’s less easy, for instance, simple things like getting blood. You can wait hours for blood or platelets at some hospitals” (S)*

*“In private it makes it more difficult because you don’t really know what the status of the patient is, because there is no ROTEM to go by” (P)*

*“Something like a research nurse that would enable the data collection and the day to day management and the education of people, I think, is probably the tool that is best suited to do it in the public sector” (A)*

*“Sometimes I can't get what I need because sometimes there are resource limitations. Sometimes specific hospitals don’t have specific products, especially the private ones that are smaller” (A)*

#### Social influences (social opportunity)

A very specific barrier participants discussed was the conflicting processes, goals, and outcome criteria from the pathology and haematology departments that had a direct impact on clinician’s ability to make change improvements.

*“Pathology and other departments who have various policies in place that make it difficult to implement change to begin with. It can be discouraging” (A)*

*“If I wanted to bring that in, I would have to sit with haematology, and they are concerned that I don’t get a rebate, or they don’t get paid enough to do platelet function tests. They are not interested in doing it because it will be cost for their department, without money for their department because the savings are in the blood bank which is a different department. That’s an external factor. That is a problem” (A)*

*“There was a resistance for a long time from haematology to basing any decisions on viscoelastic testing” (A)*

*“A haematologist who hasn’t quite got their head around that … we care about this and we don’t want to use blood products inappropriately. We don’t want to treat with a therapy that just isn't needed, not necessary. So that's our brick wall, that's our problem here, we can’t get around that” (A)*

*“Private pathology is not supporting that sort of stuff, because there is no reason really for them to do it” (A)*

Participants overwhelming recognised that effective teamwork, collaboration, and communication between disciplines was critical for successful implementation and management of bleeding.

*“I think the things most contingent on managing bleeding, are the roles and relationships between the clinicians” (P)*

*“ … because if you’ve got a bleeding patient and we’re at the operating table, I’m trying to deal with my bits, trying to fix the bleeding the ways I can, and so I rely on my anaesthetist to look at the ROTEM (Rotational thromboelastometry), interpret the ROTEM, and start to communicate what he thinks is the best way forward. It’s absolutely collaborative. It can’t be done any other way” (S)*

*“it’s a multi-disciplinary program. To me, that is absolutely fundamental for the program having success, that everyone believes that they are part of the program they have developed, and they have ownership” (A)*

*“it’s not the machines or the tests that make them (projects or programs) successful and make them right. It’s the human interactions and the protocols and the building of teams that leads to success. I think the way to overcome the barriers is to build strong teams” (A)*

### Motivation: reflective motivation, automatic motivation

‘Motivation’ (COM-B) refers to all the cerebral processes that direct behaviour, for example identifying with a professional role and evaluating potential consequences and benefits. Six barriers and five facilitators were categorised as influencing participants’ motivation to manage bleeding or implement change to improve practice. These included three each related to “Belief about capabilities”, and “Belief about consequences”, and two each related to “Social professional roles and identity” and “Emotion”.

#### Belief about capabilities (reflective motivation)

Participants perceived a lack of confidence or familiarity with change or project management limited their ability to implement improvements.

*“Unless you have them in change management is a huge, big deal. It’s not just evidence, you know, it’s leadership, it’s bringing people on board, it’s building a team behind you and getting that first four and the second four and moving forward. I think there is a lack of skill in change management. Intrinsically they’re not there” (A)*

*“You virtually need a champion really; I don’t know whether there would be resources within ######## to support this. But it’s a lot of work and it’s a lot of effort and you’ve got to drive it and you’ve got to be persistent and dogged, and those are personality traits that don’t universally exist” (A)*

*“ … in other words, if you feel as though the action you are going to take is not really going to change practice or you don’t feel like know how, or if you’re not empowered to make change then you’re less likely to even attempt to do so” (P)*

Nevertheless, participants were confident that patients received the best care they could provide considering varied contexts, settings, as well as the resources they had available.

*“So, between my anaesthetist and my perfusionist and myself, whether it’s here or ####, you sort of cover most of your bases with the resources you have. Most of the people that I’m involved with either here, or next door are pretty knowledgeable about blood and roughly where the boundaries are for treatment, no treatment, so I think most of the bases are covered” (S)*

*“It’s not a matter of going off and having a coffee and talking about it. It’s finding the best solution in the immediate term and clearly sometimes that is a compromise” (A)*

*“We’re not able to control or measure, we don’t have a TEG or ROTEM, which means intellectually we’ve got an understanding of what the problem might be, but we have to make a decision based on the clinical environment in those situations. There isn’t enough factual evidence to support every decision that you make” (A)*

However, there was consensus by anaesthetists of a balance required to keep the surgeons “happy” and supported during difficult situations when patients were bleeding and potentially unstable. It was also considered that, as surgeons were responsible for overall patient outcomes, they were accountable in a final decision-making role. Consequently, anaesthetists felt less empowered to deliver interventions.

*“At the end of the day, the surgeon’s name is on the head of the bed and an ongoing problem that we have, is when I haven’t done a ROTEM yet post-bypass that the surgeon just says, this patient is going to need platelets because they are on antiplatelets” (A)*

*“There have been times that I have just stopped transfusing. “Stop telling me it’s a coagulopathy”. It becomes a difficult position. You have got a bleeding patient and now you have got conflict and sometimes it’s about for the safety of the patient, you just have to try and move past that conflict” (A)*

*“here it’s definitely a team approach. I feel part of that team and I feel that I can use my knowledge to help come to a team decision but ultimately if a surgeon feels that there is a specific intervention that he or she would like me to do, then they are ultimately the ones managing the bleeding” (A)*

*“ … . there’s an inherent need for the anaesthetist doing the list to keep the surgeon happy. The surgeon doesn’t like the concept of blood conservation, then the anaesthetist would find it a struggle to put those things in place” (A)*

*“I try and monitor it all and bring it all together. At the end of the day, the surgeon’s name is on the head of the bed” (A)*

*“I’m not going to say to a surgeon, don’t use that product on a particular patient because they are at the point of treatment and I’m not” (A)*

*“there are some anaesthetists that work with some surgeons, mostly they follow their pattern of practice. So, it can be difficult sometimes applying all the guidelines” (A)*

#### Social professional roles and identity (reflective motivation)

Several conflicting constructs around “social professional roles and identity” were evident. These centred around drivers for change, implementation of practice improvement, and the influence of the public or private setting. Anaesthesia were primarily perceived as the drivers to change practice however, there was consensus that success was dependent on surgeon ‘buy-in’.

*“The anaesthetists are really the ones who are the overarching drivers of blood management, the surgeons seemed to have embraced that as well … well they have to, or it won’t work” (P)*

*“At an institution level, program level, I think anaesthesia probably have more involvement than surgery does” (S)*

*“It's more than just managing the bleeding, you have to manage the surgeon, perfusion, the situation, the environment as well as everything else going on with the patient. You learn this with time, some never learn” (A)*

*“If you’ve got the surgeon on board, everybody is on board in the whole process” (A)*

*“Well, intraoperatively it’s the anaesthetic team that has a primary role because we’re at the point of care. The surgeon is busy doing their highly skilled job and they are trying to stop as much bleeding as possible and minimise the amount but we’re the ones who have got the full picture at that stage. We’ve got the clinical picture. We know what lab results are. We know what the status is of blood bank. We know the status is of cell saver and perfusion” (A)*

*“I think it’s a collaborative approach, but I think it’s mainly between surgeons and anaesthetics is the two biggest ones, however, I do think anaesthetics is the one who needs to take the role. In these situations, especially stressful situations with large amounts of bleeding, the surgeon is busy. They are operating. It’s good to have their input but I think at that moment in time the anaesthetist is best suited to lead that charge” (A)*

*“I think surgeons … … … it’s hard for them because they are distracted by managing the bleeding surgically, they can’t necessarily take an overall view at the time but in terms of strategies and hospital wide policies, there is no reason why a surgeon couldn’t, here it’s just been anaesthesia, we’ve done it”(A)*

*“I do actually because I think … . there are several reasons. One is that it’s perioperative blood management. Anaesthetists are peri-op physicians, very well placed to do that. We often have a window to see our patients pre-operatively and it’s a task that we can take, that honestly the surgeons would rather we did I think, because they have got plenty of other things to do” (A)*

Participants provided insights into the social/professional differences and behaviours displayed by individuals operating in, or across the public and private sectors.

*“Where this falls down (especially in the private sector) is the working relationship that the anaesthetist has to have with a surgeon, your private work is contingent on that” (A)*

*“It’s different, in the private sector, I work with the same team all the time so it’s a bit easier because my anaesthetist has a similar approach. In the public, it’s not so easy from a staffing point of view because you’re working with different people but in public, we have a better structure for what should happen to patients” (S)*

*“In the private sector, the surgeon definitely takes the lead because there is less of a system around you to manage blood and it’s less easy, for instance, simple things like getting blood. You can wait hours for blood or platelets at some hospitals” (S)*

*“I work in a small institution that is private practice and I am responsible for my patients, so I have to take all the decisions” (S)*

#### Belief about consequences (reflective motivation)

Many participants reported differences in applying bleeding management strategies relating to private/public context with potential important consequences. Specifically, in the private sector, organisational culture meant that delaying surgery due to known bleeding risk (i.e., platelet dysfunction) was not desirable owing to financial implications, or the patient’s desire not to delay surgery.

*“There’s a lot more pressure in the private sector, even though there is documented evidence of platelet disfunction, they’ll still push for the patient to have surgery because there is a patient desire to get the surgery done, so they bleed, and they just give platelets” (A)*

*“So, the private centres are all competing with each other in order to get the surgery and the money, so they don’t want to be the one that seemed to be delaying patients” (A)*

*“Their bottom line just comes down to dollars more than anything else. It has to be seen to be costing less money, or saving money in some aspect for it to be valid” (P)*

Participants were not confident that implementing improvements bleeding management could be achieved with variable funding incentives such as the acquisition of blood products. The differences varied across states, and public and private hospitals.

*“I believe that the expense that we can take in order to prevent bleeding would be more than mitigated by the outcomes of reduced bleeding, reduced transfusions, improved patients’ haemoglobin post-operatively, the improved outcomes they would get, speedier recovery, 100%” (A)*

*“It has a cost-benefit. It’s harder to prove a cost-benefit to the hospital because we don’t pay for blood. Blood is free. Everything else costs, blood management costs, cell salvage costs money. Trying to get cell salvage in and they just said, what’s the cost benefit, how much will it cost? You try like a business case, but you can’t use blood cost as a cost. You have to find other cost savings” (A)*

*“I would hate to be at the point which will happen soon, where we’ve made most of our cost savings from changing a transfusion practice and be wanting to implement other things that we know improve clinical care but come at a cost, it’s going to be an uphill battle to try and argue for the cost, even though it’s for better patient management” (A)*

*“Bleeding has to be managed well otherwise the consequences will be poorer outcomes. That is well established” (A)*

Participants consistently reported the belief that managing bleeding with evidence-based strategies provided benefits to the patient and the organisation.

*“The other thing that is really fascinating about this, is that it’s not just we’re doing it here, its reproducible between institutions, because having fostered the same change in ##### Hospital we actually have the equivalent outcome. So, this is a reproducible process” (S)*

*“There should be an expectation now that when you walk in for an elective operation that you walk out without having to be transfused. That will be the norm and it would be unusual to be transfused if you’re doing everything in the bundle of care” (A)*

*“Because all of these blood related issues with regard to mortality and some morbidities are in the low percentages and can a long time to actually see a difference, a change or a positive benefit for patients and its only when they see it, that they can go “there is merit to this, it’s not just anti transfusion, there is patient benefit, there is hospital benefit, there is financial benefit” (P)*

*“I knew that we could do better and if we did better, then we wouldn’t have to take our patients back as much. So, that was actually what drove me to try and get a better understanding of what was going on” (A)*

“*The consequences of poor bleeding management are that we’re most likely going to have more bleeding, poorer patient outcomes, and higher costs associated with it” (A)*

### Emotion (automatic motivation)

Participants unanimously reported that they were generally not troubled by emotion related to managing actual clinical bleeding.

*“You know, a difficult case, there can be certain emotions around but that’s generally, not usually a problem” (A)*

*“Not really, not more than any other aspect of clinical care” (A)*

*“No, emotions aren't helpful in difficult clinical situations” (A)*

*“Not really. I mean, it’s part of our game” (A)*

Participants did however, report frustration that evidence-based bleeding management strategies existed, and clinicians were not able to implement these strategies to improve patient care.

*“If you can’t get buy-in from any of the other teams, from management, it’s just you, the lonely voice, it’s hard” (A)*

*“Administrative bureaucracy that prevents improvements from happening. That’s one thing you have to overcome. I think that’s definitely a big barrier, it’s frustrating” (A)*

## Discussion

This study aimed to develop a comprehensive understanding of barriers and facilitators facing cardiac surgeons, anaesthetists and perfusionists implementing improved bleeding management practice in Australian cardiac surgery units, using theoretically grounded frameworks (TDF and COM-B model). We report important individual, social, and environmental barriers influencing clinician behaviour within this complex and under-reported reality;

### Capability

- lack of confidence with change management skills
- variability with non-technical skills
- lack of cross discipline cardiac surgery specific bleeding management education

### Opportunity

- complicated institutional processes, including lack of organisational support
- lack of dedicated blood management clinicians (preferentially nursing)
- incongruent goals

### Motivation

- disparities between public and private healthcare services

Key messages for enabling successful implementation were facilitating;

### Capability

- standardisation,
- monitoring, auditing, and feedback of data,
- cross discipline training

### Opportunity

- improved interpersonal and interdepartmental collaboration through shared goals
- efficient, supportive processes to allowing clinicians to navigate unfamiliar business and financial models of health care.

Our findings suggest as individuals, clinicians generally had the motivation to make change. It might seem unlikely that ‘any’ headway to improve care can be made by health care providers in the face of organisational complexity and bureaucracy, and yet it does. This body of work demonstrates there is strength in the bottom-up approach. In fact, health care teams are creative in response to complexity, often improving patient care, ‘in spite’ of the systems in which they work. The strategies are not always elegant, rather with an approach that negotiates competing demands, organisational hypocrisies and interprofessional tensions. It is important that healthcare organisations live up to their obligation and responsibility to partner with clinicians in supporting change and improving goal directed best practice.

Many variables of the healthcare work environment including infrastructure, staffing, equipment, and other resources were perceived to have a negative influence on clinicians ‘physical opportunity’ to implement change, with variables differing between public and private hospitals. Previous literature suggests these are not unique to bleeding management in cardiac surgery, with workload and time pressures often cited barriers to behaviour change [36].

Environmental factors can go beyond those tangible variables to include complicated and unfamiliar administrative processes, lack of proficiency and fluency with the development of quality initiatives, procedures, business cases that may be required for new equipment, therapies, or additional clinical time [37, 38]. A link was revealed between ‘opportunity’ and ‘capability’ with clinicians feeling restricted by their role delivering health care, and their capability to deliver behaviour change interventions echoed in data generated in this study. The development of skills to deal with these organisational and environment issues are often not of interest to many medical professionals putting them at a disadvantage and poorly prepared to advance their change management goals [24]. Awareness of the need and ability to improve these skills may also be related to the fact that implementation science is generally published in journals that anaesthetists, perfusionists and cardiac surgeons are less likely to read.

Results from this study revealed interplay with the constructs of physical ‘opportunity’ and ‘capability’. Cross training and interprofessional education in health has been demonstrated to improve the delivery of care, a concept our participants believe would, in theory, be an important technique to improve decision-making across the surgical team [39–41]. No participants were aware of an opportunity for this type of interprofessional education in Australia. Furthermore, creating a training program incorporating surgeons, anaesthetists and perfusion was judged unlikely to occur with presence the ‘surgical’ group who were perceived to consider themselves as ‘ruggedly independent’. Indeed, there was a belief (not unanimous) that with 6 years of training, surgeons *“knew how to manage bleeding”.* It is possible surgeons tend to downplay non-technical skills, regarding group training as a waste of their time. Methods to overcome this issue are likely to involve significant negotiating skills as surgeons tend to perceive their leadership and communication skills requires no improvement [42, 43]. These are significant issues as it is clearly reported that there are more incidents of preventable harm through poor management and leadership than clinical incompetence [44].

‘Social opportunity’ also has key role to play, as it is evident that while that cardiac surgery requires the collaboration of multiple expert clinicians with specific technical skills, non-technical skills are no less important. The social environment influencing effective relationships between clinical and non-clinical departments, including the alignment of goals, and understanding of incentives and drivers, are known to be central to organisations delivering high quality care [45, 46]. This study highlighted the need for support beyond the immediate clinical team with poor alignment of goals eroding clinicians’ ‘opportunity’ to improve practice. This was particularly evident through a perceived absence of support from both public and private pathology, and haematology departments for viscoelastic haemostatic assays and platelet functions tests. These types of diagnostic assays are increasingly used to diagnose deficits in haemostatic capacity without which, treatment therapies are given ‘blind’ [47–52].

An identified lack of consistency of the team unit, particularly in public hospitals highlighted the use of standardised ways of working to improve clinician ‘capability’. Decision support tools, checklists, protocols, and procedures are well documented to reduce variation in care and improve outcomes [25, 26, 47–49, 53]. While the cardiac surgical team generally agreed with this concept; there was perceived need for autonomy in decision making. Clinical decision making is not always 'facts applied to a problem'. It was considered important to balance the use of standardisation and recommendations from guidelines (considered to be aggregated needs of the cohort) with an intuitive, experiential approach, contextualising patient problems to determine best practice for the needs of the individual patient [25, 54].

A number of explanatory themes crosslinked with ‘opportunity’ and ‘motivation’ and vice versa*.* There was a level of frustration from clinicians regarding their capacity to full embrace evidenced-based bleeding management to improve outcomes for patients, and the organisations. Implementing change (specifically in the private sector) was complicated by the perception of pressure for surgery (despite risk of bleeding and potential requirement for blood transfusion) from; 1. patients to get surgery done, and 2. the organisation *not* to cancel surgery. ‘Motivation’ for change was further complicated by the view from anaesthesia of a requirement to ‘keep surgeons happy’ to ensure ongoing private work. These issues are in conflict with the mandatory requirement to implement bleeding management strategies to achieve Australia’s National Safety and Quality Health Service Standards [55]. While these standards have been judged to drive organisational bleeding management change ‘at a minimum’, they were not considered to drive excellence [25, 26, 49, 54].

While there is currently little qualitative research on facilitators to implementing bleeding management in cardiac surgery in Australia, two studies have recently reported the benefits of monitoring, audit, and feedback of data on outcomes and performance to reduce variability in practice [25, 26]. This is also demonstrated in studies across other cohorts which also suggest capitalising on the driven and competitive behaviour of surgeons through regular analysis and feedback of data on outcomes and performance can be utilised as enabler to improve practice [56–60].

#### Limitations

The results from this interview study should be interpreted in the context of the combination of strengths and limitations innate with all research**.** While a broad cross-section of participants from varied hospitals and States/Territories were interviewed, findings may not have captured all relevant themes. Of note, operating room nurses were not included as they do not make decisions regarding the management of bleeding. However, as nurses are in the same physical space as the included participants during the management of intra-operative bleeding, a lack of contribution from this group may be seen as a limitation. Additionally, participants’ observations and interpretations are subjective, and they may be biased in their viewpoints. However, the different interpretations and perceptions are undoubtedly part of the phenomena of this unique dynamic. Further, the involvement of three researchers in the analysis improves the trustworthiness and credibility of the analysis; and the use of theoretically grounded frameworks facilitates comparisons between contexts, enabling theoretical generalisability. Strengths of this study include the variety of beliefs included member-checking to inform validity, and the achievement of data saturation across themes early in the analysis.

## Conclusion

This study describes how factors associated with capability, opportunity and motivation were perceived by cardiac surgeons, anaesthetists and perfusionists and their ability to implement improvements in bleeding management practice. The findings suggest that while the knowledge and skills of this team are vast, the inclusion of the wider team has a significant impact. Although an obvious overall goal of those directly and indirectly involved would be positive patient outcomes, when specific aims are identified, not everyone’s targets are aligned. Individual clinician barriers were identified as a lack of confidence with change management skills, variability with non-technical skills, lack of cross discipline cardiac surgery specific bleeding management education, complicated institutional processes, lack of dedicated blood management clinicians, incongruent goals, and disparities between public and private healthcare services. Key messages for enabling successful implementation were facilitating practice improvements were standardisation, monitoring, auditing, and feedback of data and efficient, supportive processes to allowing clinicians to navigate unfamiliar business and financial models of health care. What is clear from this study is that no one strategy can improve practice; success is dependent on ‘mixing and matching’ improvements of technical and non-technical skills and procedural and organisational measures, in conjunction with commitment to overarching shared goals. We now know, what needs to be done to support clinicians to close the knowledge practice gap; the next step is to formulate opportunities to make it happen.

## Supplementary Information

**Additional file 1.** Interview Topic Guide for Cardiac Surgeons, Cardiac Anaesthetists, and Perfusionists.

**Additional file 2.** TDF coding manual.

## Data Availability

The datasets used and/or analysed during the current study are available from the corresponding author on reasonable request.

## Abbreviations

ANZCA: Australia and New Zealand College of Anaesthetists
ANZSCTS: Australian & New Zealand Society of Cardiothoracic Surgeons
COM-B: Capability, Opportunity, and Motivation Behaviour Model
CPD: Continuing professional development
PBM: Patient Blood Management paradigm
ROTEM: Rotational Thromboelastometry
TDF: Theoretical Domains Framework
WHO: World Health Organisation
