# Supplementary material for: Barriers and facilitators to implementing evidence based bleeding management in Australian Cardiac Surgery Units: a qualitative interview study analysed with the theoretical domains framework and COM-B model
Source: BMC Health Serv Res. 2021 Jun 5;21:550. doi: 10.1186/s12913-021-06269-8 (PMC8178922; doi:10.1186/s12913-021-06269-8)
Supplement: Supplementary file 1 — Additional file 1. Interview Topic Guide for Cardiac Surgeons, Cardiac Anaesthetists, and Perfusionists. [file 12913_2021_6269_MOESM1_ESM.docx]

**Additional File 1**

**Interview Topic Guide for Cardiac Surgeons, Cardiac Anaesthetists, and Perfusionists.**

Introduction: The aim of the interview is to help us understand and explain what influences clinicians when they make decisions regarding strategies that may influence a patient’s risk of bleeding or when patients are actually bleeding.

There are no right or wrong answers; we are trying to understand how clinicians from different groups approach this issue; including societal / governance / or organisational influences and the impacts of those influences, both locally and externally, the clinical environment, the influence of colleagues, personal motivations etc……

No identifiable data will be included,….

So just some basic questions to start:

What type of setting do you practice in? (Metropolitan Public or Private, Regional Public or Private

How many years have you practiced in total, not just your speciality?

How would you describe the bleeding management strategies you consider, or typically use, when you are delivering care to your cardiac surgery patient pre-operatively and intra-operatively? (Environmental Context)

The next lot of questions are more questions about what influences your decisions regarding bleeding management strategies, what you think about, when you are delivering care to your patient pre-operatively and intra-operatively. Some questions may seem repetitive, but the questions are derived from different theories of human behaviour and we are trying to figure out which theories best apply in this area.

Specifically, The questions are around “your pre-op assessment of bleeding risk and peri-op management of bleeding for patients undergoing cardiac surgery” –

And by cardiac surgery, I’m referring to all patients having cardiac surgery from elective to emergency aortic dissection to transplant;

Are you aware of any guidelines (institutional, national, international) on bleeding management in cardiac surgery? Which ones do you follow?

Do you think guidelines are useful or a waste of time? Explain

Do you use the guidelines to direct your practice? (If so which ones?)

How important do you think guidelines are? (who do you think they are important for?)

What other evidence do you use to direct practice?

If you think there is not enough quality evidence, how do you make decisions on how to manage bleeding?

Do you feel you have the authority to make decisions and the freedom to act in accordance your **professional** knowledge base on bleeding management?

Do you believe your practice to be fully evidence-based, to the extent that it could be when you consider the evidence you are aware of? How could it be improved

Are you aware of any specialised training on bleeding management for clinicians working with cardiac surgery patients?

How much expertise, education or experience do you think clinicians needs to have, to effectively manage bleeding?

How much influence do external factors affect your ability as a clinician to implement what you believe is evidence-based bleeding management?

Are there any competing tasks, time constraints, available tools / or lack of… that might influence whether or not you dedicate time to a pre-op evaluation for bleeding risk?

Are there aspects of your clinical environment (physical vs. resource factor) that influence whether or not you feel you are able to implement best evidence-based strategies to manage bleeding?

What would help you overcome these problems/difficulties? (Prompt: skills training in medical curriculum, communication techniques, formal training programs, CME, educational material on line or by mail)

How easy or difficult is it for you personally implement strategies you believe are evidence-based, to manage bleeding risk or actual bleeding? (Prompt: Management, finance, health service, pathology etc)

How easy or difficult is it for you personally to call a diagnosis for example surgical bleed or coagulopathy?

Are you confident that you are able diagnose a bleeding as surgical / coagulopathy or a combination of both?

Would any other team members influence whether or not you implement certain bleeding management strategies for your patients having cardiac surgery? (Prompt: who else; Other clinicians; medical staff including nurses and residents/fellows; relatives; surgeons contribute to the management of bleeding risk / actual bleeding)

How might the views/opinions of others, affect you implementing strategies to management bleeding risk / actual bleeding?

Do your colleagues generally agree with you on how to manage bleeding risk / actual bleeding? Can you give examples?

Do patient sentiments influence the strategies you use to manage bleeding in cardiac surgery? Explain

Does your ability to assess bleeding risk or manage actual bleeding evoke worry or concern in you? How so?

Do you believe managing bleeding, is cost effective / or have a cost benefit in cardiac surgery? Why or why not

What do you think the consequences of not having the ability to provide evidence-based bleeding might be; both positive and negative? (prompt: to yourself, patients, to colleagues, short and long term)

What would be an incentive for you to apply evidence-based strategies that you believe exist, but don’t apply (if this is the case at your institution? (Prompt: goals within yourself? external?)

What would you, personally, have to do to improve the way you manage bleeding at your institution?

Follow-up; In an ideal world, where anything is possible, what would see as best process for assessing bleeding risk or managing actual bleeding?

If you wanted to implement changes in your own practice (individual/team setting/practice setting) to improve the assessment of bleeding risk or managing actual bleeding, what would be some ways to do this? (Prompt: Role of Protocol or guidelines)

That’s all the questions I have for you; has anything occurred to you about this topic that we haven’t asked about?

Thank you
