# Supplementary material for: Barriers and facilitators to implementing evidence based bleeding management in Australian Cardiac Surgery Units: a qualitative interview study analysed with the theoretical domains framework and COM-B model
Source: BMC Health Serv Res. 2021 Jun 5;21:550. doi: 10.1186/s12913-021-06269-8 (PMC8178922; doi:10.1186/s12913-021-06269-8)
Supplement: Supplementary file 2 — Additional file 2. TDF coding manual. [file 12913_2021_6269_MOESM2_ESM.docx]

**TDF coding manual**

| **Domain Definition** | **Constructs** | **Ask yourself questions** | **Notes** |
| --- | --- | --- | --- |
| **COM – B………. Automatic Motivation** | | | |
| **Emotion**  (A complex reaction pattern, involving experiential, behavioural, & physiological elements, by which the individual attempts to deal with a personally significant matter or event) | **Anxiety / Frustration** | Do you feel anxious or fearful when you are managing bleeding? | - Satisfying - Frustration - Feelings or affect about using evidence-based BM (e.g. feel anxious about patient’s situation & therefore revert to empirical treatment) |
|  | **Fear. Worry** | Do you feel additional stress? |  |
| **Reinforcement**  (Increasing the probability of a response by arranging a dependent relationship, or contingency, between the response & a given stimulus) | **Consequents Reinforcement** | Will imposing restrictions or prescriptive requirement improve BM? | - Monitoring or perception of (lack of) monitoring - Whether any incentives, positive or negative consequents influence behaviour on managing bleeding |
| **COM – B………. Physical Opportunity** | | | |
| **Environmental context & resources**  Any circumstance of a person's situation or environment that discourages or encourages the development of skills & abilities, independence, social competence & adaptive behaviour | Environmental stressors | Are there competing time constraints? | - Describing the presence or absence of tools /resources / equipment/ services/ / clinicians / organisational structures which facilitate/impede performing the behaviour….ie. - Decision support tools, algorithms - Lack of equipment/space - Lack of clinicians to support POCCT - Poor/inappropriate training - Clinical demands - Lack of time - Describing how the organisational practice / culture / impedes performing BM - Factors related to the setting /environment /person-environmental interactions that influence behaviour about managing bleeding (physical, things..) - Examples of the environmental context and resources in this study could be the availability of support services, equipment, service-level pathways of care for BM, staffing levels, whether organisation culture prioritises/provides resource for BM or not etc |
|  | Clinical Resources / Material Resources | To what extent does the availability of physical or resource (including clinicians) factors affect the ability to manage bleeding? |  |
|  | Organisational culture | Do overarching policies, procedures, management / finance staff support or not support bleeding management? |  |
|  | Barriers & Facilitators | Are there other barriers & facilitators to BM? |  |
| **COM – B………. Psychological Capability** | | | |
| **Behavioural regulation**  Anything aimed at managing or changing objectively observed or measured actions | Self-monitoring | Self-monitored behaviour where clinicians make an effort to change or regulate themselves; reflecting an ability to modify one's behaviour in response to situation | - Statements where clinicians want audit/evaluation/feedback on their BM - Statements about processes in place/needed to monitor BM / outcomes of BM - Statements about prompts / processes etc used or required to make the behaviour sustainable / routine / habit - Ways of doing things that relate to pursuing & achieving desired goals, standards or targets that could influence the management of patients with bleeding - Translating intention into action (e.g. at the individual level – action planning; at the organisational level – changing / introducing specific processes related to bleeding management) - Statements about using conscious effort to ensure the behaviour is carried out: I plan in advance/make notes/use prompts, so I don’t forget to…. - In the context of this study, behavioural regulation may relate primarily to the need of algorithms, decision support tools, apps, standard procedures relating to BM |
|  | Breaking habits | BM requires breaking well-formed habits |  |
|  | Standardisation | BM requires a plan of action. Tools. Procedures (standardisation) |  |
|  | Feedback | BM and outcomes of BM need to be monitored |  |
| **Knowledge**  An awareness of the existence of something | Knowledge (including knowledge of BM / scientific rationale | Do they know the guidelines? What do they think the guidelines say or mean, or what the evidence is behind them? | - (Lack of) specific BM training* - Existing procedural knowledge - Knowledge/use of guidelines/evidence (knowing they exist & believing they are high quality) - Knowledge about bleeding management / physiology / pathophysiology - Statements about having/not having/wanting factual or procedural knowledge of when & how to do the behaviour - Statements about having/not having/wanting an understanding of the rationale behind performing the behaviour - I know/do not know/want to know how/when to do the behaviour - I know/do not know/want to know why I should do the behaviour - In the context of this study, knowledge of the condition/scientific rationale could relate to knowledge of bleeding management strategies, risks of bleeding, etc. - Knowledge of these factors may be both correct & incorrect knowledge. |
|  | Procedural knowledge of BM  environment | Do they know what they should be doing to manage bleeding & why they should be doing it |  |
|  | Knowledge of the task environment | What does the environment require? What is needed in the environment to manage bleeding effectively? |  |
|  | Knowledge Education and Training | Has knowledge been acquired from formal education? Are you involved in providing BM education? |  |
| **Memory, attention & decision processes**  (The ability to retain information, focus selectively on aspects of the environment & choose between two or more alternatives) | Memory/ Attention / attention control | BM is something they remember to think about / include in their decision making. | - The processes involved, the factors (patient / context), taken into account when making BM decisions - Forget/remember about specific BM strategies - Use environmental, social or internal stimuli to prompt or cue performance of wanted behaviour or non-performance of unwanted behaviour |
|  | Cognitive Input | How much cognitive power is required? |  |
| **Skills (physical, cognitive & interpersonal)**  An ability or proficiency acquired through practice | Skills | Do they know how to do it? | - Competence, ability, proficiency (diagnosing the cause of/managing bleeding) - Using guidelines & evidence-based strategies to manage bleeding - Previous experience/training - Statements about wanting to develop/improve skills in managing bleeding   - In practice, I do x,y,z to help me with managing bleeding   - I would like more training in how manage bleeding   - We haven’t had any training in how to manage bleeding - In the context of this study, skills may be interpersonal skills (e.g. practical advice, promote benefits, non-judgemental approach, normalisation, communication skills etc). |
|  | Skill development | Have they had the right training? |  |
|  | Competence | Do they feel competent in managing bleeding? |  |
|  | Ability | How easy or difficult is it to manage bleeding in the given context? |  |
|  | Interpersonal skills | Do they have the necessary interpersonal skills to work with other to manage bleeding? |  |
|  | Practice | Are there adequate opportunities to practice? |  |
|  | Skill assessment | Are they assessed & given feedback on the quality of their management? |  |
| **COM – B………. Reflective Motivation** | | | |
| **Beliefs about capabilities**  (Acceptance of the truth, reality, or validity about an ability, talent, or facility that a person can put to constructive use) | Perceived competence | What would help to improve their management of bleeding? | - Perceptions about his/her own competence/self-confidence in managing bleeding (e.g. very confident) - Perceptions about control over his/her own behaviour, i.e. whether managing bleeding is within or out with his/her control - Self-efficacy – a person’s confidence that he/she can employ the skills that are necessary to cope with stress, & mobilise resources required to meet the demands of the bleeding situation. The person’s own internal attribution of their ability to do this………. - Ease or difficulty of implementation/ change/ improvement of BM - Statements of healthcare professional’s confidence, judgements about their competence and control in their ability (or inability) to manage bleeding   - I do/don’t feel confident/ able/ capable/ competent to manage bleeding   - I find it difficult/easy etc to manage bleeding   - I feel that I have/don’t have control to manage the bleeding - In the context of this study, beliefs about capabilities relates to clinicians making evaluative judgments on their ability to manage bleeding for example their confidence in being able to have a rational discussion with other team members - This would also include expressing optimism/pessimism of effectively discussing BM |
|  | Self-efficacy | How difficult or easy is it for them to manage bleeding? |  |
|  | Perceived behavioural control | Do they have enough control to manage bleeding? |  |
|  | Beliefs | Do they believe they can effectively manage bleeding? |  |
|  | Professional confidence | Do they believe they have the skills, ability to manage bleeding? |  |
| **Beliefs about consequences**  (Acceptance of the truth, reality, or validity about outcomes of a behaviour in a given situation) | Outcome expectancies | Characteristics of the cognitive, emotional and behavioural outcomes that individuals believe either promote or inhibit effective BM | - Perceptions about outcomes, & advantages & disadvantages of evidence-based bleeding management - Statements relating to healthcare professionals’ beliefs/views etc on the - outcome/consequences of doing/not doing the behaviour - Statements can include positive or negative consequences of doing/not doing the behaviour   - If I do/don’t do the behaviour, x,y,z will happen   - Doing the behaviour will have a beneficial/adverse impact on me/my patient - I do/don’t do x, y, z because otherwise x, y, z will/will not happen |
|  | Anticipated regret | A sense of the potential negative consequences of decisions related to BM |  |
|  | Consequences | An outcome of decision related to management of bleeding |  |
| **Goals**  (Mental representations of outcomes or end states that an individual wants to achieve) | Goal priority and target setting | Are there goals set in the immediate or distant future related to BM? | - What stage of change with BM are clinicians at? - Goals, priorities, importance, commitment to a certain course of actions or behaviours |
|  | Implementation intention | Has a plan to improve BM been put into action? |  |
| **Intentions**  (A conscious decision to perform a behaviour or a resolve to act in a certain way) | Stability and stages of intention | How strong is their intention to improve BM (Not considering, considering, acting) | - Is bleeding management a paradigm all clinicians will have to engage in? |
| **Optimism**  (The confidence that things will happen for the best or that desired goals will be attained) | Optimism | Are they optimistic about the outcome? | - The confidence expressed that things will happen appropriately (clinically) i.e.   - that bleeding will be managed successfully   - that BM will be implemented successfully   - that clinicians will work together collaboratively - Pessimism also coded within this domain i.e. managing bleeding will end in disaster on weekends, when certain staff are not available - BM will not be implemented as a standard of care - Collaboration will not be achieved |
|  | Pessimism | Are they pessimistic about the outcome? |  |
| **Social/professional role & identity**  (A coherent set of behaviours & displayed personal qualities of an individual in a social or work setting) | Professional identity | What do they think about the credibility of the source of the guidelines? | - Clinicians’ expressions about their own professional identity/job/role/professional boundaries & comparisons about their role with that of other professionals (e.g. managing bleeding treating is part of my role within the team) - Personal physical activity beliefs/identity - (Not) others’ role/responsibility - Implementation/change/improving BM is their responsibility (or not) |
|  | Professional Role / Confidence | Is the BM within the role of the professional? How confident are they about BM despite the difficulties? |  |
|  | Social identity | Do they think a guideline should determine their behaviour? |  |
|  | Identity | Do they identify with BM? |  |
|  | Professional boundaries | Is BM compatible with professional standards? |  |
|  | Group identity | Do they think their BM is in keeping with what others are doing? |  |
|  | Leadership | Management or leaders facilitate the delivery of BM (or not) |  |
|  | Organisational commitment | Management or leaders are willing to listen to problems associated with BM or are resistant to supporting change |  |
| **COM – B………. Social Opportunity** | | | |
| **Social influences**  **(**Those interpersonal processes that can cause individuals to change their thoughts, feelings, or behaviours) | Group consensus | Different clinical groups or individuals who have an impact on BM or implementing BM strategies are supportive / not supportive, invested /not invested, or have a different focus or perspective, can’t agree | - Supportive others - Mentorship (i.e., sharing resources; champion) - Priority/value at group level - Generic support (i.e., not resources) - Social pressure (The exertion of influence on a person or group by another person or group) - Social norms (Socially determined consensual standards that indicate   - what behaviours are considered typical in a given context   - what behaviours are considered proper in the context - Power/hierarchy (The capacity to influence others, even when they try to resist this influence) - Intergroup conflict - Disagreement or confrontation between two or more groups & their members. - Group identity consensus - The set of behavioural or personal characteristics by which an individual is recognisable [& portrays] as a member of the group - Organisational culture/climate (A distinctive pattern of thought & behaviour shared by members of the same organisation & reflected in their language, values, attitudes, beliefs & customs) - Organisational development - Leadership (The processes involved in leading others, including organising, directing, coordinating & motivating their efforts toward achievement of certain group or organisation goals) - Team working |
|  | Social norms | Most individuals in their clinical group think that BM is important/ not important |  |
|  | Modelling | Others clinical groups model appropriate/inappropriate behaviours with respect to BM |  |
|  | Power | The power balance / hierarchy with different clinicians and clinical groups affects BM, or the ability to implement or improve or manage bleeding |  |
|  | Teamwork | Members of a group feel collaboration when managing bleeding is important / not important  Practicing/not practicing BM forms an important part of group identity |  |
|  | Conflict | Conflicts within a group or between groups affects the delivery of effective BM |  |
|  | Organisational Culture/Climate | Do all clinicians / clinical groups share the same ideals / goals/ beliefs about BM |  |

Coding manual based on definitions provided in Cane, O’Connor & Michie (2012). TDF, Theoretical Domains Framework
